# Molecular characterization of *Pseudomonas* sp. isolated from lower respiratory tract infection in HIV and non-HIV population by *16S* rDNA and ARDRA

**DOI:** 10.1186/1471-2334-14-S3-P17

**Published:** 2014-05-27

**Authors:** C Anitha, Sujatha Kabilan, N Rajinish, A Santhosh Kumar, Padma Krishnan, Illaikiam Rasikan, S Senthilkumar, S Vincent, S Senthamarai, S Sivasankari, P Gunasekaran, Rajasekharan Sikhamani, M Pushkala

**Affiliations:** 1 Dept of Microbiology (Faculty of Medicine), Dr. ALM PGIBMS, University of Madras, Taramani, Chennai, India; 2 School of Biological Science, Madurai Kamaraj University, Madurai-21, India; 3 SRM College, Chennai, India; 4 LIFE/P.G and Research Department of Advanced Zoology and Biotechnology, Loyola College, Chennai, India; 5 Dr.K.M.Cherian Heart Foundation and frontier Lifeline Pvt. Ltd, Elavur, Gummidipoondi-601201, India; 6 Department of Microbiology, Meenakshi Medical College and Research Institute, Enathur, Kanchipuram, India; 7 Government Hospital of Thoracic Medicine, Tambaram Sanatorium, Tambaram, Chennai, India; 8 Department of Immunohaemotology, Dr. M.G.R Medical University, Guindy, Chennai, India

## Background

*P. aeruginosa* an important pathogen causing lower respiratory tract infections (LRTI) both in HIV and non-HIV population. Molecular characterization of *Pseudomonas* spp. helps in better understanding of their clonal distribution among these patient populations. Our study aims to discriminate and generate highly specific fingerprints using 16S-rDNA PCR and amplified ribosomal DNA restriction analysis (ARDRA) techniques.

## Methods

Seventy-two isolates (45-HIV, 24-Non-HIV, 2-environmental, 1-ATCC) of *Pseudomonas* spp. were subjected to 16SrDNA PCR using universal primers and amplicons digested with *Hae III*, *Alu I and Rsa I* for ARDRA analysis. Nucleic acid size confirmation of the digested amplicons was done using MultiNA Bioanalyser. Phylogenetic tree was constructed by maximum parsimonious method using MEGA 4.0. Representative isolates from the major clones were sequenced and submitted to genbank for accession numbers and genetic relatedness was identified by UPGMA using NTSYS 1.80 software. Mean genetic distance (GD) and intraspecies mean GD were calculated.

## Results

Based on ARDRA banding pattern, 14 groups and 10 clones were obtained from the 72 pseudomonas isolates with the following accession numbers: JF279962-64, JF303639-45. Sequences showed 97-100% similarity with known *P. aeruginosa*, *P.putida*, *P.stutzeri*, *Alcaligenes feacalis* strains by BLASTN analysis. The overall mean GD and intra-species GD of the various species was as follows: *P. aeruginosa* (JF279962, JF279963, JF303643, JF279964, JF303645, JF303644): 4.664, 16.663, 7.536, 0.733, 0.096, 2.402 and 0.221, 3.487, 2.010, 0.029, 2.728, 0.011;*P.putida*(JF303640, JF303639):11.904, 7.3 and 1.064, 1.371; *P.stutzeri* (JF303641) is 2.732, 0.036; *Alcaligenes feacalis* (JF303642) : 30.248, 5.377 respectively.

## Conclusion

The highly variable mean GD values amongst the isolates from different and within the same species indicates high genetic diversity among *Pseudomonas* spp causing LRTI among HIV and Non HIV patients.

